# Body Mass Index and Brain Aging: Microstructural Effects Mapped with Advanced Diffusion MRI

**DOI:** 10.1002/alz.093338

**Published:** 2025-01-09

**Authors:** Sebastian M Benavidez, Katherine E Lawrence, Leila Nabulsi, Emily Laltoo, Zvart Abaryan, Julio E Villalon‐Reina, Talia M Nir, Iyad Ba Gari, Alyssa H Zhu, Elizabeth Haddad, Neda Jahanshad, Paul M. Thompson

**Affiliations:** ^1^ Imaging Genetics Center, Mark and Mary Stevens Neuroimaging & Informatics Institute, University of Southern California, Marina del Rey, CA USA; ^2^ Children's Hospital Los Angeles, Los Angeles, CA USA

## Abstract

**Background:**

High body mass index (BMI) is a risk factor for dementia, and prior diffusion‐weighted magnetic resonance (dMRI) work has shown that higher BMI is associated with lower white matter (WM) integrity. The tensor distribution function (TDF) is an advanced dMRI model that is sensitive to the effects of both healthy aging and Alzheimer’s disease (Nir et al., 2017; Lawrence et al., 2021). Here we use TDF – together with the conventional dMRI model diffusion tensor imaging (DTI) and the advanced dMRI model neurite orientation dispersion and density imaging (NODDI) (Zhang et al., 2012) – to characterize BMI effects on WM microstructure aging in later adulthood in the large‐scale UK Biobank dataset.

**Method:**

Cross‐sectional dMRI data from 34,763 participants were analyzed (64.7 ± 19.5 years old, 48.4% male) from the UK Biobank dataset. WM metrics were extracted for 20 WM regions of interest (ROI) using the ENIGMA‐DTI protocol and FSL’s tract‐based spatial statistics (TBSS) (Jahanshad et al., 2013; Smith et al., 2006). We examined the interaction between BMI and age (demeaned linear and quadratic age terms) while including standard nuisance covariates. Results were corrected for multiple comparisons across ROIs using the false discovery rate method.

**Result:**

Significant interactions were observed between BMI and age across the deep WM for every dMRI model examined (all corrected *p*<0.05). When averaging across the full WM, higher BMI was significantly associated with lower fractional anisotropy (DTI‐FA) among older participants (corrected *p*=0.04). Orientation dispersion (NODDI‐OD) was relatively higher in those with higher BMI in midlife coupled with less pronounced higher values in older adults (corrected *p*=0.03). Higher BMI was also associated with higher isotropic volume fraction (NODDI‐ISOVF) in middle‐age (corrected *p*=0.02).

**Conclusion:**

These results in a cross‐sectional sample reveal that BMI is significantly associated with age trajectories in older individuals’ brain WM microstructure. This may help elucidate the biological mechanisms of BMI as a risk factor for dementia. Future research should consider longitudinal cohorts that include individuals who convert to dementia, to better understand how BMI affects WM aging.

